# Microstructural Origin of the High-Energy Storage Performance in Epitaxial Lead-Free Ba(Zr_0.2_Ti_0.8_)O_3_ Thick Films

**DOI:** 10.3390/ma15196778

**Published:** 2022-09-30

**Authors:** Jun Ouyang, Xianke Wang, Changtao Shao, Hongbo Cheng, Hanfei Zhu, Yuhang Ren

**Affiliations:** 1 Institute of Advanced Energy Materials and Chemistry, School of Chemistry and Chemical Engineering, Qilu University of Technology (Shandong Academy of Sciences), Jinan 250353, China; 2 Shandong Industrial Ceramics Research and Design Institute, Zibo 255031, China; 3 Physics and Astronomy, Hunter College, The City University of New York, New York, NY 10065, USA; 4 The Graduate Center, The City University of New York, 365 5th Avenue, New York, NY 10016, USA

**Keywords:** ferroelectric films, polymorphic phase boundary (PPB), domain structure, lead-free, strain engineering

## Abstract

In our previous work, epitaxial Ba(Zr_0.2_Ti_0.8_)O_3_ thick films (~1–2 μm) showed an excellent energy storage performance with a large recyclable energy density (~58 J/cc) and a high energy efficiency (~92%), which was attributed to a nanoscale entangled heterophase polydomain structure. Here, we propose a detailed analysis of the structure–property relationship in these film materials, using an annealing process to illustrate the effect of nanodomain entanglement on the energy storage performance. It is revealed that an annealing-induced stress relaxation led to the segregation of the nanodomains (via detailed XRD analyses), and a degraded energy storage performance (via polarization-electric field analysis). These results confirm that a nanophase entanglement is an origin of the high-energy storage performance in the Ba(Zr_0.2_Ti_0.8_)O_3_ thick films.

## 1. Introduction

Ferroelectrics with a composition near a morphotropic or polymorphic phase boundary (MPB or PPB) often exhibit anomalous functional responses under the stimulation of an electrical or mechanical field, such as Pb(Zr_1−*x*_Ti*_x_*)O_3_ (PZT), (1-*x*)Pb(Mg_1/3_Nb_2/3_)O_3_−*x*PbTiO_3_ (PMN−PT), (K,Na)NbO_3_ (KNN) and Ba(Ti_0.8_Zr_0.2_)O_3_−*x*(Ba_0.7_Ca_0.3_)TiO_3_ (BZT-BCT) [1,2,3,4,5]. A chemical substitution in the above-mentioned material systems is an effective way to drive the MPB- or PPB-like behavior with large piezoelectric or ferroelectric responses. These responses are commonly attributed to the shallow energy barriers between the multi-polar state ferroelectric phases [6,7]. Under an external field, these shallow energy barriers could be overcome easily, leading to phase transformations and strongly enhanced functional properties [8,9,10]. Recently, Pan et al. constructed polymorphic nanodomains in epitaxial ferroelectric films by combining two regular ferroelectrics with a paraelectric component. By disrupting the long-range polar order of a homogeneous ferroelectric, this design has created a relaxor-like, multi-polar ground state at the nanometer scale, leading to a greatly enhanced energy storage density [11]. However, due to a random mixing of the ferroelectric nanophases, a sizable remanent polarization (*P*_r_) still remains, which hinders the improvement of the charge–discharge efficiency of ferroelectric-based multi-layer or film capacitors [11,12].

Compared to the chemical substitution, engineering of an epitaxial strain (“strain engineering”) was demonstrated to be an alternative effective way to drive the MPB- or PPB-like behavior with enhanced ferroelectric responses [13,14,15,16,17]. Zeches et al. used an epitaxial strain to drive the formation of an MPB consisting of a metastable pseudo-tetragonal (T) and a stable pseudo-rhombohedral (R) phase in BiFeO_3_ thin films [17]. Compared with films consisting of pure R or T phase, BiFeO_3_ films with a mixture of T and R phases show a much higher piezoelectric response [18]. Recently, polymorphic nanodomains with coexisting tetragonal-like (T) and orthorhombic-like (O) phases were reported in BaTiO_3_ films grown on (110) GdScO_3_ substrates, due to the effect of an asymmetric misfit strain [19]. Polymorphic nanophases could also be created by engineering the mechanical constraint from the underlying substrate [19,20,21,22]. Strain-relaxed epitaxial BaTiO_3_ films grown on (111)-SrTiO_3_ substrates displayed coexisting R, O and T phases in the form of nanodomains ranging from 1–10 nm [21]. Meanwhile, coexisting T and R phases were discovered in CVD-processed PZT thick films (~1.5 μm) on (111)-SrTiO_3_, which was believed to be the reason for a large piezoelectric strain [21]. 

Landau free energy profiles which are characterized by a shallow energy well together with a small energy difference between multi-polar states may be easily fine-tuned by applying a doping or substitution method on simple perovskite ferroelectrics [23]. A desirable strain-induced heterophase polydomain structure could be realized in the as-grown film. The electric polarization of such a film can closely follow the variation of field strength, resulting in a linear-like *P-E* loop [24,25,26]. Substituting Ti^4+^ with Zr^4+^ in BaTiO_3_ will slightly enlarge its unit cell, reduce its self-polarization and draw its multipolar ground states closer to each other, resulting in a large number of polymorphs in epitaxially strained films [25,26]. In our previous work, 1.4 μm thick epitaxial Ba(Zr_0.2_,Ti_0.8_)O_3_ films displayed a pseudo-linear, ultra-slim *P-E* hysteresis loop with a large recycle energy storage density (~58 J/cm^3^) and efficiency (~92%) [27]. Here, by utilizing a thermal annealing process, we present a focused analysis of the structure–property relationship in the Ba(Zr_0.2_,Ti_0.8_)O_3_ films. The XRD and *P-E* hysteresis results indicated that a relaxation of the misfit stress via the annealing process has led to the segregation of the nanodomains and a degraded energy storage performance. It is concluded that the excellent energy storage properties of the Ba(Zr_0.2_Ti_0.8_)O_3_ thick film are mainly due to its entangled heterophase nanodomain structure.

## 2. Materials and Methods

**Materials.** (100)-oriented SrTiO_3_ (STO) single crystal and SrRuO_3_ sputtering targets were prepared by Anhui Institute of Optics and Fine Mechanics (Chinese Academy of Sciences, Hefei, China). The Ba(Zr_0.2_Ti_0.8_)O_3_ (BZT) ceramic target, with the same shape and size as those of the SrRuO_3_ target (*Φ* = 50 mm, t = 5 mm), was prepared by a conventional solid-state reaction method. X-ray diffraction measurements of the BZT target revealed a single rhombohedral phase with a lattice parameter of ~4.06 Å.

**Deposition of the BZT Film.** BZT films with a thickness from 350 nm to 1.25 μm and a SrRuO_3_ bottom electrode of ~100 nm thick were sequentially deposited on STO(100) substrates, in an RF-magnetron sputtering system with a base pressure of 2.0 × 10^−4^ Pa. The SrRuO_3_ bottom electrode and BZT film were both sputtered in a mixed Ar/O_2_ atmosphere (Ar:O_2_ = 4:1) at a deposition pressure of 1.2 Pa and a substrate temperature of 650 °C. After deposition, the as-grown film was kept at 650 °C for 10 min and then cooled down to room temperature at a rate of 7–8 °C/min with a pure O_2_ of 1.2 Pa. Although, the annealed samples also experienced the same procedures, the only difference was that these films were kept at 650 °C/1 h in pure O_2_ atmosphere with different pressures (1.2 Pa and 10 Pa). The sandwiched metal–ferroelectric–metal (MFM) structures were prepared by sputtering circular Au dots (*Φ* = 200 μm) on the surface of the BZT film at a low-pressure chamber via a shadow mask.

**Characterizations.** The phase structures and crystallographic orientations of the BZT films were characterized by X-ray diffraction (XRD) using a Dmax-rc diffractometer (Toyo, Japan) for 2*θ* scans and a Smart Lab Rigaku for pole figures measurements. TEM (Transmission Electron Microscopy) in a JEM-2010 microscope (JEOL, Tokyo, Japan) and STEM (Scanning Transmission Electron Microscopy) in an ARM-200CF (JEOL, Tokyo, Japan) were used to investigate the nanoscale polymorphic phase structures. A Focused Ion beam milling technique was applied to prepare the cross-sectional TEM/STEM samples (Scios2, FEI, Waltham, MA, USA). The ferroelectric hysteresis loops (*P-E* loops) and the leakage current characteristics (*I*-*V* curves) of the BZT films were measured by using an RT-Precision LC ferroelectric tester (Radiant Technology, Redmond, WA, USA), at a frequency of 1 kHz. 

## 3. Results

**Figure 1:**
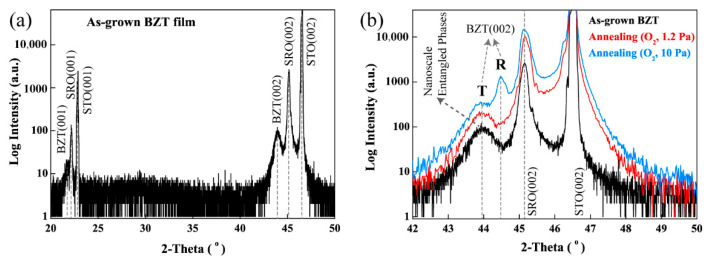
XRD 2*θ*-scan patterns of (**a**) the as-grown BZT film (1.25 μm thick); (**b**) the BZT films annealed at 650 °C and in a pure O_2_ (1.2 Pa or 10 Pa) atmosphere, in comparison with that of its as-grown state.

Figure 1a shows a representative XRD 2*θ*-scan pattern of the as-grown BZT films. A single perovskite phase with only {00*l*} diffraction peaks indicates a highly c-axis-oriented growth of the BZT films on an SRO(100)/STO(100) substrate. Figure 1b is the XRD 2*θ*-scan patterns of as-grown and annealed BZT films in a pure O_2_ atmosphere for 1 h (with different annealing oxygen pressures: 1.2 Pa and 10 Pa). It is noted that the film composition will not change after the annealing process as there are no volatile elements in the film. The full width at half maximum (FWHM) of the (002) diffraction peak for the as-grown film is wider than those of the other two annealed films. Using the *d*_(002)_ spacing (2.057 Å) from the XRD 2*θ*-scan pattern of the as-grown film and that of the ceramic target of BZT (*d*_(002)_~2.0285 Å), and through the Poisson’s effect, the residual in-plane strain is estimated to be about ~−1.9% for the 1.25 μm thick BZT film [28]. In addition to a significant shift with respect to the bulk (002) peak, the broad FWHM of the (002) peak suggests that the BZT film may have a nanoscale entangled heterophase structure with its majority component being a T-like phase (with a longer out-of-plane axis). Such a strain-induced heterophase nanostructure was verified through its relaxation after annealing (in a pure O_2_ atmosphere at 650 °C for 1 h). In Figure 1b, the annealed BZT films displayed a narrow (002) peak and an elevated right shoulder (near the dashed line which marks the position of the bulk (002) BZT peak). Specifically, in the BZT film annealed in the high O_2_ pressure, the broad (002) peak split into a rhombohedral peak and a tetragonal one. These dramatic changes in the (002) BZT peak indicate that thermal annealing has induced segregation of the entangled nanophases, as well as a transformation from the majority of T-like phases into the stable bulk rhombohedral phase.

**Figure 2:**
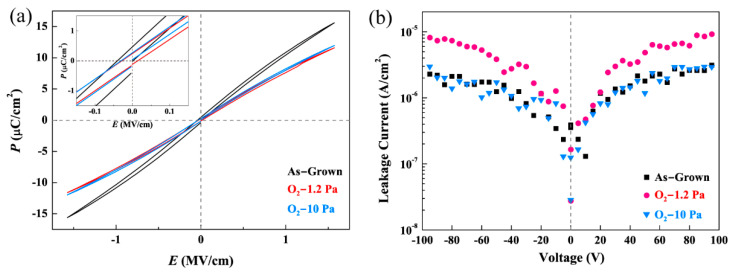
The room temperature *P-E* hysteresis loops (**a**) and leakage current density–voltage curves (**b**) of the as-grown and annealed BZT films (in 1.2 Pa and 10 Pa O_2_).

The *P-E* hysteresis loops of the as-grown and annealed BZT films are shown in Figure 2a at the same applied electric field. Smaller *P*_r_ (0.26 μC/cm^2^ @ 10 Pa, 0.29 μC/cm^2^ @ 1.2 Pa), *P*_max_ (11.6 μC/cm^2^ @ 10 Pa, 12.0 μC/cm^2^ @ 1.2 Pa) and lower coercive field (inset of Figure 1a) values are shown by the annealed films, in comparison with those of the as-grown film (*P*_r_: 0.49, *P*_max_: 15.6 μC/cm^2^). These changes in the *P-E* characteristics of the annealed films can be attributed to nanophase segregation and a substantial transformation of the phase structure from the T-like to the R-like ones, as shown in Figure 1b. As is discussed in our previous work, the rhombohedral phase has a hierarchical polytwin structure with {100}-type domain boundaries. Under charging/discharging electric fields, such a structure shows a lower coercive field and smaller polarization values than those of the tetragonal ones [24,29]. Additionally, in Figure 2b, in comparison with that of the as-grown film, the annealed film in a 1.2 Pa O_2_ atmosphere shows a higher leakage current, while an almost identical leakage current behavior was shown by the film annealed in a 10 Pa O_2_ atmosphere. The increased leakage current in the low O_2_ pressure annealed BZT film may be attributed to an increased amount of diffusional defects resulting from the high-temperature process. On the other hand, the recovery of a low leakage current in the film annealed at a higher O_2_ pressure (10 Pa) can be explained by a reduction of the oxygen vacancies in the film, which was processed at a lower O_2_ pressure (1.2 Pa) prior to the annealing. These results in XRD and hysteresis measurements were similar with our previous work in fatigued samples [30].

**Figure 3:**
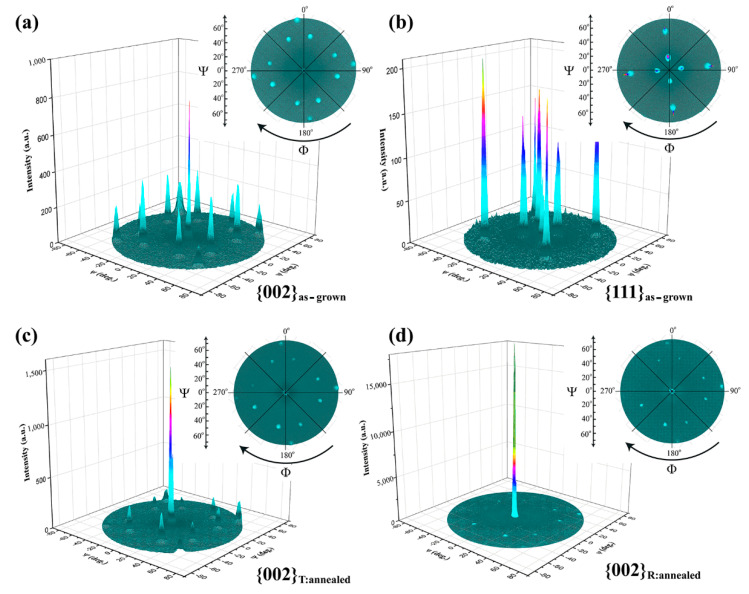
XRD pole figures (**a**,**b**) using the (**a**) {002} and (**b**) {111} diffraction peaks of the as-grown BZT film; (**c**,**d**) the split (**c**) {002}_T_ and (**d**) {002}_R_ peaks of the annealed BZT film (in 10 Pa O_2_); (insets: top view of of (**a**–**d**)).

The XRD pole figure measurements using the (002) and (111) BZT peaks are conducted for a detailed analysis of the nanoscale entangled T/R phases (“heterophase nanodomains”) in the as-grown film (Figure 3a,b). The central peak near Ψ = 0° comes from the (002) diffraction of the nanoscale entangled T and R phases in the as-grown film, while the well-regulated dots (Ψ~48° and ~71°) in the film represent three-fold {221} oriented T-phase nanodomains. Eight small peaks at Ψ~48° can be divided into two groups, corresponding to {2 1 2}- and {2 −1 2}-oriented nano T-phases, which are separated by an *Φ* angle of ~35°. This measured angle difference is a little smaller than the calculated one (~38.9°), which may be due to a cubic lattice approximation, and a small rotation of the nanophases under the effect of the film–substrate misfit strain. The four peaks at Ψ = ~71° are {221}-oriented nano T-phases. Polarizations of {2 1 2}-, {2 −1 2}- and (2 2 1)-oriented nano T-phases are aligned along a direction of ∼48°, ~48° and ~71° away from the film normal, respectively. Meanwhile, in Figure 3b, the (111) pole figure displays four peaks at Ψ~17° and Ψ~54.7°, suggesting the coexistence of {221}- and (001)-oriented nanophases in the BZT film. The volume ratios of the (001) and {221}-oriented nanophase structures were estimated by comparing their integrated XRD intensity counts (I_(002)_/I_{221}_) from the pole figures. The as-grown film showed a I_(002)_/I_{221}_ phase ratio of ~3.75:1 (Intensity Counts: 306,958_(002)_, 81,764_{221}_) from the (002) pole figure, and ~3.45:1 (Intensity Counts: 36,781 _(002)_, 10,661_{221}_) from the (111) pole figure. These results suggest that the volume ratio of the {221}-oriented T-like nanophases was ~20% in the as-grown BZT film. It is noted that the {221}-oriented T-like nanophases contributed to the broad diffraction peak near (003) BZT in the XRD 2*θ*-scan pattern [27]. 

As discussed in Figure 1b, the broad (002) peak corresponding to nanoscale entangled T/R phases splits into two peaks, (002)_T_ and (002)_R_, after thermal annealing in a pure oxygen atmosphere of 10 Pa at 650 °C for 1 h. The post-annealing microstructure is investigated via pole figure analysis of the annealed BZT film, using its (002)_T_ and (002)_R_ diffraction peaks (Figure 3c,d). The integrated XRD intensities of the (002) (Ψ~0°) and the {221} (Ψ~48° and Ψ~71°) diffractions are 286,270_(002)-3c_, 2,972,960_(002)-3d_, 59,294_{221}-3c_ and 22,355_{221}-3d_ counts, obtained from the (002)_T_ (Figure 3c) and (002)_R_ (Figure 3d) pole figures, respectively. Compared with those from the as-grown BZT film, the above results of the annealed BZT film reveal that: (i) the intensity counts increased substantially, which confirms an improved crystalline quality of the BZT film (Intensity Counts ratio at Ψ = 0°: ~10.6) as a typical annealing effect; (ii) the integrated XRD intensity ratio of {221}_3c_: {221}_3d_ is ~2.7, which verifies that the {221}-oriented nanostructure is a tetragonal phase; and (iii) the R-like phase in Figure 3d showed a 10-times higher integrated XRD intensity than that of the T-like phase (I_R(002)-3d_/I_T(002)-3c_ = ~10.4), indicating that the R-like phase is dominant in the annealed film. This is consistent with the result of Figure 1b.

**Figure 4:**
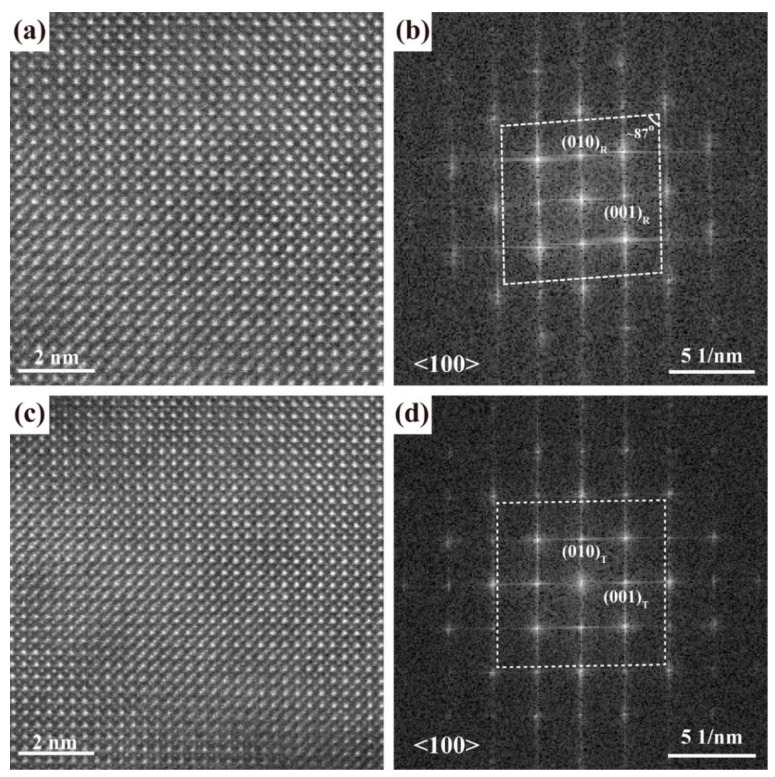
STEM analysis. Atomic scale HAADF-STEM images from (**a**) an R phase region and (**c**) a T phase region of the as-grown BZT film. Corresponding electron diffraction patterns via FFT are shown in (**b**,**d**) for the T and R phases, respectively.

Nanoscale entangled T/R phases of the as-grown BZT film are analyzed via high angle angular dark-field (Z-contrast) scanning transmission electron microscopy (HAADF-STEM, Tokyo, Japan). The representative HAADF-STEM atomic images of the rhombohedral and tetragonal-like phases, together with their electron diffraction patterns obtained by FFT (Fast Fourier Transition), are shown in Figure 4. The parallelogram in Figure 4b displays an angle of ~87° between the (0*l*0) and (00*l*) planes, confirming a rhombohedral symmetry for the lattice structure shown in Figure 4a. On the other hand, the diffraction pattern in Figure 4d is rectangular-shaped, verifying a tetragonal symmetry for the lattice structure shown in Figure 3c. Although these R- and T-like phases were observed via STEM and TEM [24] throughout the film, they could not always be observed by regular XRD 2*θ*-scans due to the low resolution of the XRD method in probing the nanostructure of an epitaxial thick film, especially when there is a nanoscale entanglement of phases with close lattice parameters.

**Figure 5:**
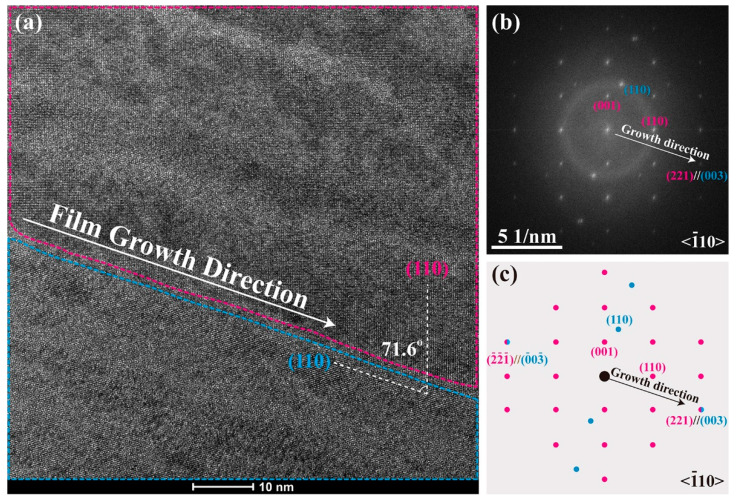
(**a**) A high-resolution TEM image of the as-grown BZT film with a <−1 1 0> zone axis near the bottom electrode, (**b**,**c**) are an FFT electron diffraction pattern from (**a**) and a schematic drawing for the diffraction pattern (blue dots: {00*l*}_R_ phase, red dots: (221)_T_).

Figure 5a–c show a high-resolution TEM image and its FFT diffraction pattern, as well as a schematic diffraction pattern for the T_(221)_/R_(001)_ heterophase nanodomain structure in the as-grown BZT film, respectively. The high-resolution TEM image displays an {00*l*}_R_//(221)_T_-type crystalline orientation. The angle between (221)_T_ plane and a {110}-type T/R phase boundary (marked red and blue in the T and R phase regions, respectively) is ∼73.5° (vs. ∼71° from consideration of a cubic structure), as is shown in Figure 5a. The FFT diffraction pattern in Figure 5b shows the coexistence of T_(221)_/R_(001)_ nanophases, which is schematically shown in Figure 5c. The diffraction spots of T_(221)_ and R_(003)_, both along the film growth direction, overlapped with each other due to a close match of their ((221) and (003)) *d*-spacings.

## 4. Discussions

In the nanoscale entangled T/R phases in the BZT thick film, the misfit strain induced coexisting R- and T-like phases with an {*l*00} out-of-plane orientation and a {110} interface, as shown in Figure 4, are the backbone or matrix of the heterophase nanodomain structure across the film thickness. Meanwhile, the scattered {221}_T_ nanophases help to mediate the interphase misfit between the {*l*00}-oriented R- and T-like phases. These heterophase nanodomains contributed to the strong central spot in (002) pole figures of the as-grown and annealed BZT films (Figure 3a,c,d). Meanwhile, the (221)-oriented nanodomains, with a three-fold orientation, in the film bulk are the “in-plane” dominated T-phases, and their polarizations are aligned ~71° away from the film normal. On the other hand, the “out-of-plane” and “in-plane” polarization components for the (2 1 2) and (2 −1 2)-oriented T-phase nanodomains are equal due to their polarizations aligned along a direction of ~48° away from the film normal. The effects of these nanophase structures on the energy storage performance are: (1) the entangled {*l*00}-oriented T/R nanodomains in the as-grown BZT film will make the polarization switching much easier and polarization saturation much harder, resulting in a slim *P-E* loop with a small P_r_ and a delayed polarization saturation. Such a *P-E* loop corresponds to a large recyclable energy density and a high charge–discharge efficiency; (2) the {221}-oriented T-phase nanodomains, scattered in the matrix of the entangled T/R polydomains, led to a net in-plane polarization. This results in a smaller remnant polarization of the BZT film (measured out-of-plane), further improving its energy efficiency. Lastly, a large poling field is necessary to completely align these nanodomains, at which the electric polarization and energy storage density of the film are significantly improved.

## 5. Conclusions

The μm thick BZT films which feature a slim *P-E* hysteresis loop and a delayed polarization saturation displayed a large recyclable energy density and a high charge–discharge efficiency. These excellent energy storage properties are attributed to a nanoscale entangled heterophase polydomain structure. The structure–property relationship was investigated by employing XRD, TEM and STEM techniques, as well as a high-temperature annealing process. It was revealed that the nanoscale entangled T/R phases are segregated following a relaxation of the film stress via annealing. The annealed film possesses a slimmer *P-E* loop with lower *P*_r_ and *P*_max_ values, corresponding to a degraded energy storage performance. The XRD pole figure and TEM analyses confirmed the coexistence of {*l*00}_R,T_ and {221}_T_ heterophase nanodomains in the as-grown and annealed films. The film structure can be described as an entangled {*l*00}-oriented T/R phase matrix with scattered {221}_T_ nanophases. The entangled {*l*00}_T,R_ nanodomains can be switched much easier and saturate much harder under an external electric field. Meanwhile, the {221}_T_ nanophases led to a net in-plane polarization. Overall, such a heterophase nanodomain structure features a pseudo-linear *P-E* loop with a small *P*_r_, a delayed *P*_max_ and a high breakdown field, resulting in a greatly improved energy storage performance [19,22].

## Data Availability

Not applicable.
